# Novel Structural Insight into Inhibitors of Heme Oxygenase-1 (HO-1) by New Imidazole-Based Compounds: Biochemical and In Vitro Anticancer Activity Evaluation

**DOI:** 10.3390/molecules23051209

**Published:** 2018-05-18

**Authors:** Khaled F. Greish, Loredana Salerno, Reem Al Zahrani, Emanuele Amata, Maria N. Modica, Giuseppe Romeo, Agostino Marrazzo, Orazio Prezzavento, Valeria Sorrenti, Antonio Rescifina, Giuseppe Floresta, Sebastiano Intagliata, Valeria Pittalà

**Affiliations:** 1 Department of Molecular Medicine, College of Medicine and Medical Sciences, Princess Al-Jawhara Centre for Molecular Medicine, Arabian Gulf University, Manama 329, Bahrain; khaledfg@agu.edu.bh (K.F.G.); Alzahrani.reem11@gmail.com (R.A.Z.); 2 Department of Drug Sciences, University of Catania, V.le A. Doria 6, 95125 Catania, Italy; eamata@unict.it (E.A.); mmodica@unict.it (M.N.M.); gromeo@unict.it (G.R.); marrazzo@unict.it (A.M.); prezzave@unict.it (O.P.); sorrenti@unict.it (V.S.); arescifina@unict.it (A.R.); vpittala@unict.it (V.P.); 3 Department of Chemical Sciences, University of Catania, V.le A. Doria, 95125 Catania, Italy; 4 Department of Medicinal Chemistry, College of Pharmacy, University of Florida, Gainesville, FL 32610, USA; s.intagliata@cop.ufl.edu

**Keywords:** HO-1, HO-2, HO-1 inhibitors, imidazole, docking studies, in silico profiling, ADME-toxicity

## Abstract

In this paper, the design, synthesis, and molecular modeling of a new azole-based HO-1 inhibitors was reported, using compound **1** as a lead compound, in which an imidazole moiety is linked to a hydrophobic group by means of an ethanolic spacer. The tested compounds showed a good inhibitor activity and possessed IC_50_ values in the micromolar range. These results were obtained by targeting the hydrophobic western region. Molecular modeling studies confirmed a consolidated binding mode in which the nitrogen of the imidazolyl moiety coordinated the heme ferrous iron, meanwhile the hydrophobic groups were located in the western region of HO-1 binding pocket. Moreover, the new compounds were screened for in silico ADME-Tox properties to predict drug-like behavior with convincing results. Finally, the in vitro antitumor activity profile of compound **1** was investigated in different cancer cell lines and nanomicellar formulation was synthesized with the aim of improving compound’s **1** water solubility. Finally, compound **1** was tested in melanoma cells in combination with doxorubicin showing interesting synergic activity.

## 1. Introduction

A family of enzymes named heme oxygenase (HO) finely tunes the amount of heme in our body [1]. Heme oxygenase catalyzes the ring opening of protoporphyrin IX, with a simultaneous release of an equimolar amount of Fe^2+^, carbon monoxide (CO), and biliverdin (BV), which promptly transforms into bilirubin (BR) [2]. The HO family consists of two major isoforms: HO-1 and HO-2. The latter, HO-2 (37 kDa), is a non-inducible constitutive form [3]. It is predominantly represented in the brain where it is thought to be the major source of CO. Literature reports on the endogenous role of HO-2 are limited in number and further studies are needed. In non-stressful conditions, HO-1 (32 kDa, alternative name: heat shock protein 32, Hsp32) is found in tissues at low concentrations with a major presence in the spleen and liver. However, by being a powerful inducible isoform HO-1 is highly expressed under different stressful conditions including oxidative stress (OS). HO-1 upregulation by means of heme removal and CO and BV production exerts a significant cytoprotective effect and its upregulation is regarded as beneficial in conditions such OS-based diseases [4,5,6]. At a transcriptional level, the nuclear factor (erythroid-derived 2)-like 2 (Nrf2) pathway regulates, among others, the expression of HO-1 [7]. Conversely, HO-1 and HO-2 inhibition are considered a valuable anticancer approach [7,8,9]. Upregulation of HO-1 has repeatedly been reported in many types of human malignancies, and in these clinical cases, poor outcomes were reported [9]. The prooncogenic effects of HO-1 seem related to its stimulatory effects on angiogenesis, cell survival, OS protection, and to the regulation of inflammation and immune response [10,11]. Additionally, HO-1 seems to be correlated with the onset of chemoresistance to commonly used anticancer therapies.

**Figure 1:**
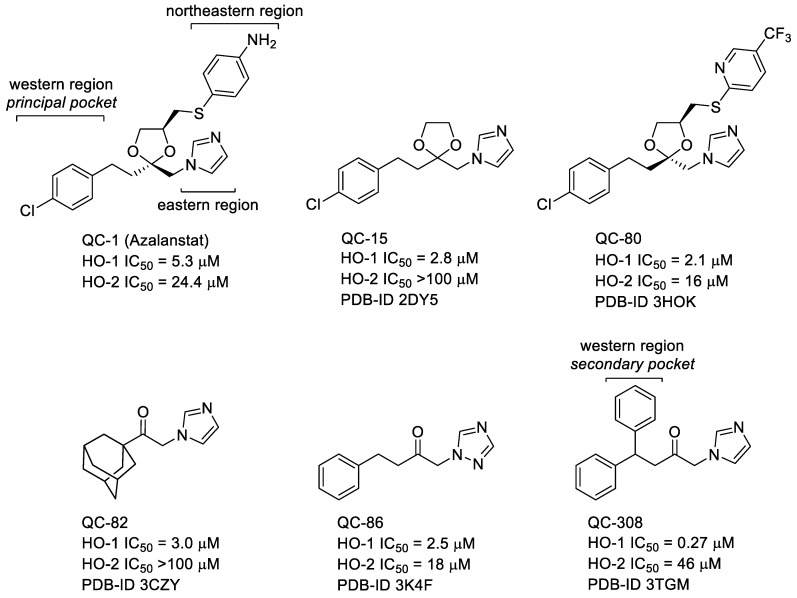
Chemical structure of HO-1 inhibitors used for X-ray crystal studies in complex with HO-1.

Metalloporphyrins (MPs) represent the first generation of HO-1 inhibitors. However, due to their strong chemical similarity with heme, they are able to interfere with other heme-containing enzymes (e.g., cytochromes (CYPs) P450 and nitric oxide synthase (NOS)) [12,13,14]. This major limitation hampered their further development. The second generation of HO-1 inhibitors, starting from Azalanstat (QC-1, Figure 1), is represented by the so-called azole-based HO-1 inhibitors [15]. Detailed structure-activity relationship (SAR) studies on this lead compound, along with co-crystallization data reported for the azole-based compounds QC-15, QC-80, QC-82, QC-86, and QC-308 complexed with HO-1, opened a way to solve the issue related to interferences with other heme-related enzymes (Figure 1) [16]. This class of compounds is commonly characterized by a peculiar non-competitive binding mode in which heme ferrous iron oxidation and O_2_ binding are prevented when heme is placed in the HO-1 binding pocket. These inhibitors contain three main features: (1) a hydrophobic portion; (2) a connecting chain of different lengths; and (3) an azole-based moiety that coordinate the Fe^2+^ atom of heme when heme is inside the HO-1 heme binding pocket [17].

**Figure 2:**
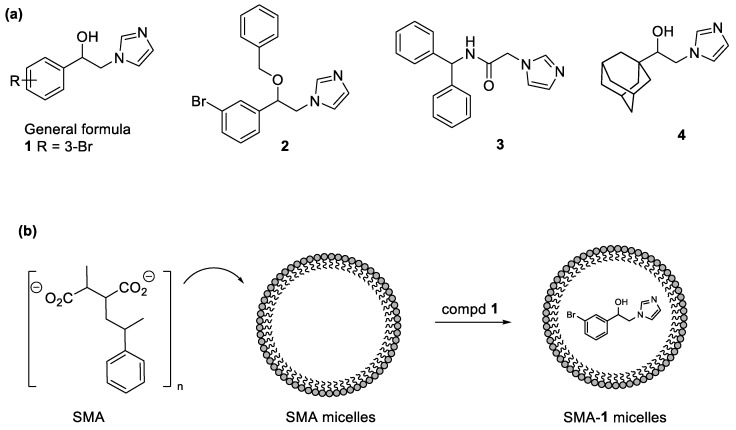
(**a**) Chemical structure of HO-1 inhibitors, **1**–**4**; (**b**) Styrene-maleic acid (SMA) micelles formation.

Our research interest is related to the development of new antitumor compounds and more specifically selective HO-1 and mixed HO-1/HO-2 inhibitors [18,19,20,21,22,23,24]. To this extent, we constructed a ligand database with more than 400 molecules (HemeOxDB, http://www.researchdsf.unict.it/hemeoxdb), similar to the one we built for sigma-2 receptor ligands [25,26], that incorporates the entire amount of published HO-1 and HO-2 inhibitors [27,28]. Recently, we reported on a novel class of HO-1 selective and HO-1/HO-2 mixed inhibitors characterized by a phenylethanolic chain linking an imidazole ring to different hydrophobic residues obtained by potholing of the hydrophobic HO-1 western region (Figure 2, General Formula) [21]. This work culminates in the finding of Compound **1** (Figure 2a), a phenylethanolic azole-based inhibitor bearing a bromine atom in the phenyl ring that represents one of the most potent HO-1 inhibitors known to date, with sub-micromolar inhibitory activity against HO-1 and moderate activity against HO-2 (HO-1 IC_50_ = 0.4 µM, HO-2 IC_50_ = 32.0 µM). The relative hydrophobicity of the compound can pose a practical challenge for further utilization in clinical settings. Furthermore, as HO-1 is a ubiquitous molecule involved in an array of physiological functions, it is advantageous to devise a targeting system to deliver the HO-1 inhibitors to the site of action (i.e., tumor tissues). To this end, we further encapsulated our lead compound into a nanodelivery system based styrene-maleic acid (SMA) micelles. Micellar encapsulation would improve the water solubility and enhance local tumor concentrations by virtue of the enhanced permeability and retention (EPR) effect [29].

We herein describe the biological evaluation of our lead Compound **1** and its SMA-micellar formulation on different cancer cell lines (Figure 2, SMA-**1**). Additionally, we tested compound **1** in melanoma cells in combination with doxorubicin. Finally, we here describe the design, synthesis, and the inhibitory potency for HO-1 of new derivatives **2**–**4**, together with their docking studies and in silico absorption, distribution, metabolism, elimination (ADME) toxicity.

## 2. Results and Discussion

### 2.1. Design

The azole-based inhibitors share a common non-competitive binding mode. Indeed, within the HO-1 enzyme, as previously evidenced by SARs and crystallographic data, at least three main crucial areas for inhibitor binding have been identified [16]. The so-called eastern region is able to allocate the azole ring that represents the first anchoring point by establishing a coordination binding to the heme ferrous iron. The northeastern and western regions, located in the proximity and in the distal area, respectively, form two hydrophobic pockets able to stabilize further the inhibitor binding and account for potency and selectivity. The western region forms a large flexible chamber and a proximal subsidiary small cavity able to allocate hydrophobic moieties of variable measures (e.g., aryl, heteroaryl, or adamantyl) [16,17]. Proper occupation of both principal and secondary pockets of the western region afforded QC-308, a double-clamp ligand, and seem to be a good strategy to improve the potency of our compounds. To this extent, we designed compounds **2** and **3** with the aim of occupying the secondary binding pocket of the western region of HO-1 (Figure 2). Compound **2** is the benzylated analog of 1; while compound **3** is a close analog of QC-308. We selected compound **1** as the lead compound for its potency, and we encapsulated it into a nanodelivery system based on SMA micelles. Nanosized carrier’s formulation (Figure 2) ensured a selective accumulation at the site of action due to the well-known EPR effect [30]. Indeed, limited lymphatic draining and tumor vasculature wide fenestration promoted selective accumulation and retention of the nanoparticles into the cancerous tissue. Finally, querying the HemeOxDB we noticed that derivatives possessing an alkyl-alcoholic connecting chain were generally stronger HO-1 inhibitors than the corresponding ketones [31,32,33]. With this in mind, we designed compound **4**, a close analog of QC-82, in which the ketone function was reduced to an alcoholic one (Figure 2).

Docking analysis and ADME toxicity in silico prediction were reported. We finally tested the most interesting compound **1** for its cytotoxic activity towards a panel of tumoral cell lines, namely MDA-MB-231, MCF-7, DU145, PC3, LnCap, and B16. In addition, a combination of compound **1** and doxorubicin was tested in B16 cells. Finally, we prepared SMA-**1** micelles with the aim of improving the water solubility of the compound.

### 2.2. Chemistry

**Scheme 1:**
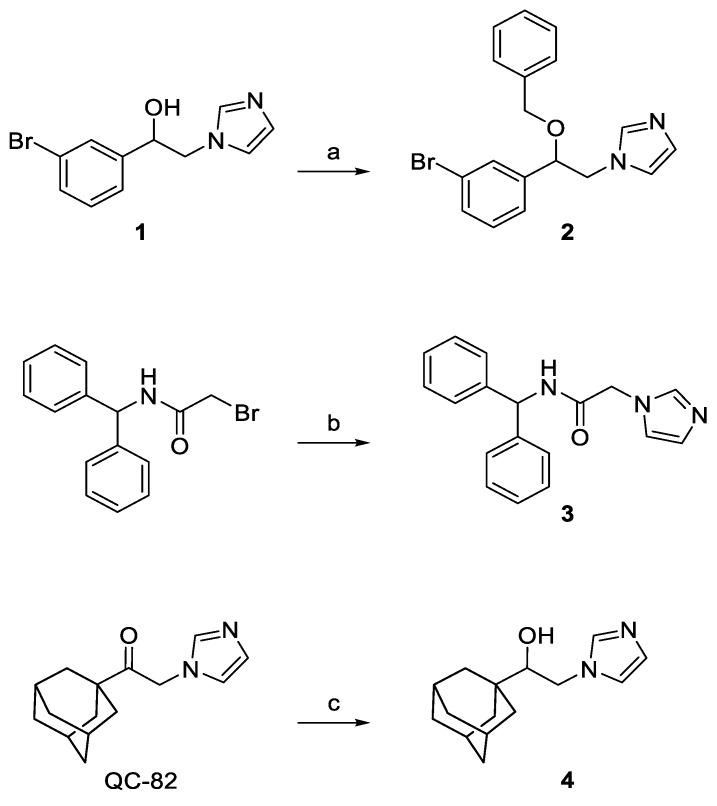
Reagents and conditions: (**a**) benzyl bromide, NaH 80% dispersion in mineral oil, dry DMF, 22 °C, 2 h, then methanol; (**b**) imidazole, K_2_CO_3_, dry DMF, room temperature, 2 h, then water; and (**c**) NaBH_4_, CH_3_OH, 2 h, reflux, then HCl 1 N, 0.5 h, reflux.

Compounds **2**–**4** were synthesized according to the general pathway illustrated in Scheme 1. Briefly, compound **1** was treated with NaH in the presence of benzyl bromide and dry DMF as solvent to afford 2 [34,35]. Compound **3** was synthesized by nucleophilic displacement of the commercially available *N*-benzhydryl-2-bromoacetamide using an excess of imidazole. QC-82 was reduced with NaBH_4_ affording the final compound **4** in high yield [36]. Compounds **2** and **4** are racemic mixtures.

### 2.3. Characterization of SMA-***1*** Micelles

The SMA-1 micelles, synthesized as reported in the experimental section, had a recovery of 80%. Recovery was calculated as the percentage of recovered material (i.e., lyophilized powder) to the total starting material. The micellar system had >30 mg/mL water solubility. The loading was 18% and is expressed as a weight percentage of 1 in the final micelles compared to the total weight of recovered SMA micelles. For targeting through the bloodstream, it is generally known that appropriate size of carriers is from ca. 10 to 200 nm in diameter [37]; the mean diameter of the SMA-**1** micelle was 180.6 ± 12.3 nm, as determined by dynamic light scattering. The polydispersity index (PDI) of SMA-1 micelles was 0.211, and the zeta potential was −0.11 mV in deionized water (Table 1). The PDI measures the size distribution relative to the mean peak, PDI < 0.3 is usually accepted in nanoformulations. The relative narrow distribution of the micelle ensures consistent biological pharmacokinetic (PK) results in further biological testing in vivo. The near neutral charge reported here is inductive of safety. Highly charged nanoformulations can randomly activate biological systems such as coagulation cascades, complement systems, platelets, and immune cells, which may result in detrimental toxicity. Neutral and near neutral charged particles are hence of valuable biological value in terms of its predicted safety [38].

### 2.4. HO-1 Inhibition and Cytotoxicity Activity

Inhibition activity assay for HO-1 was performed by extracting the enzyme from the rat spleen microsomal fraction. HO-1 activity was determined by measuring the formation of BR using the difference in absorbance at 464–530 nm, as described in the experimental section. Inhibition activity data are expressed as IC_50_ (μM) and obtained results are outlined in Table 2, utilizing Azalanstat as a reference compound. The novel synthesized derivatives, quite unexpectedly, exhibited moderate inhibitory potency towards HO-1, and accordingly we tested only the most promising compound **3** for inhibitory activity towards HO-2. Compound **2** demonstrated to be around 200 fold less potent than the unsubstituted parent compound **1**. Compound **3**, possessing an IC_50_ = 28.8 μM, revealed to be the most potent among the newly reported HO-1 inhibitors. However, compound **3** showed to be more potent toward HO-2 (IC_50_ = 14.4). Compound **4**, a close analog of QC-82 in which the ketone function has been reduced to an alcoholic one, showed unsatisfactory HO-1 inhibitory activity. The interactions of compounds **2**–**4** with HO-1 are discussed in the docking studies and revealed an agreement with the experimental data.

Taking into account HO-1 overexpression in many cancers and basing on the antitumor properties previously reported for some HO-1 inhibitors [20,39], we focused our attention only on compound **1** for further biological assay since compounds **2**–**4** revealed to be less interesting. Indeed, compound **1**, as previously reported, showed a favorable in silico ADME toxicity profile, non-tumorigenic or non-irritant effects, and without negative effects on the reproductive system [21]. Given these premises, compound **1** has been tested for its cytotoxic activity towards a panel of tumor cell lines: prostate (DU145 and PC3) and breast (MDA-MB-231) cancer hormone-resistant, and sensitive (LnCap and MCF-7, respectively). In murine melanoma (B16) cell line compound **1** was tested alone and in combination with doxorubicin. Moreover, compound **1** has been tested on the non-cancerous human embryonic kidney (HEK) cell line selected as example of healthy cells. All the selected cell lines share a moderate to high expression of HO-1, with the exception of PC3 whose HO-1 concentration is poor to moderate [40,41,42,43,44]. Finally, SMA micelles loaded with compound **1** has been tested.

**Figure 3:**
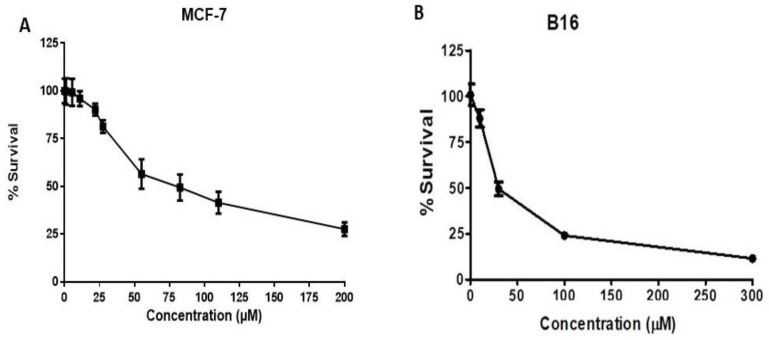
Cytotoxicity of compound **1** against MCF-7 and B16 cells. (**A**) MCF-7 (hormone-sensitive breast cancer cells) and (**B**) B16 (melanoma) cells were treated with 1.1–200 μM of compound **1** for 72 h. Control wells were treated with vehicle only (0.2% DMSO). At the end of treatment, cell number was determined using the sulforhodamine B assay. Data are expressed as mean ± SEM (*n* = 3). Non-linear regression and IC_50_ values determination were performed using GraphPad Prism 6.

Cytotoxicity data are reported in Table 3 and Figure 3. With respect to breast cancer cell lines, compound **1** showed the most interesting results for hormone-sensitive cell lines (MCF-7 IC_50_ = 52.55 μM; Table 3 and Figure 3, Panel A) when compared to hormone-resistant (MDA-MB-231 IC_50_ = 82.60 μM; Table 3). Concerning prostate cancer, compound **1** showed comparable results toward hormone-sensitive LnCap, DU145, and PC3 cell lines, in which moderate cytotoxicity was observed. Compound **1** tested towards the B16 cell line showed the best inhibitory activity (IC_50_ = 37 μM; Table 3 and Figure 3, Panel B).

**Figure 4:**
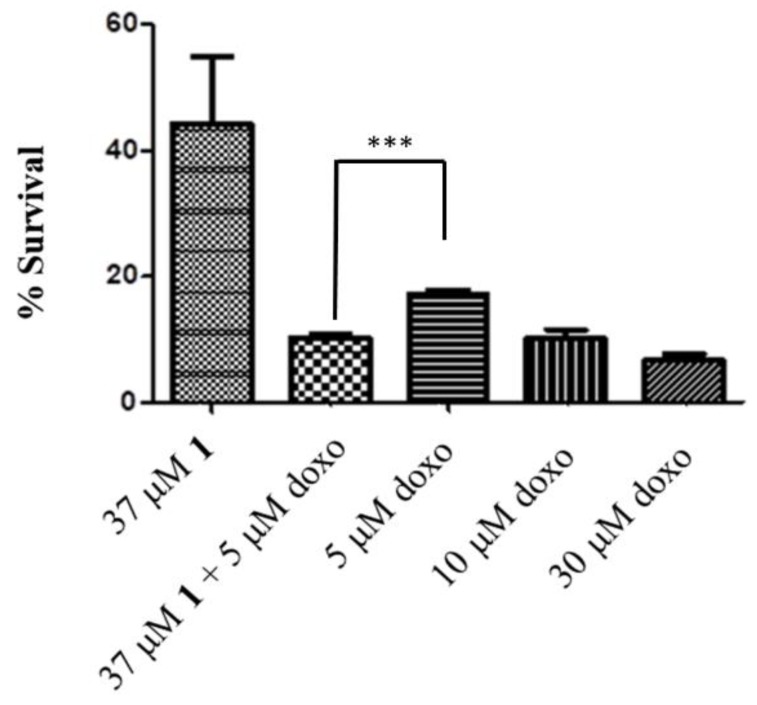
Cytotoxicity of compound **1** in combination with different concentration of doxorubicin (doxo) against B16 cells. Data are expressed as mean ± SEM (*n* = 3). *** Significant vs. untreated control cells: *p* < 0.001.

With the aim of studying potential synergistic effects, compound **1** was tested in B16 cell in combination with doxorubicin. The latter, besides acting as a DNA intercalator agent, exerts its effects through the production of reactive oxygen species (ROS) and consequent onset of OS [45]. Concomitant HO-1 inhibition might increase OS and in turn increase doxorubicin activity with consequent reduction of the dosage. Indeed, combining compound **1** (10 μM) with doxorubicin (5 μM) produced a synergistic cytotoxic effect (Figure 4). Compound **1** resulted 2–10 fold selective for cancer cells with respect to healthy cells.

Encapsulation of compound **1** into SMA demonstrated reduced cytotoxic activity. This result is quite consistent with the usual lower activity shown in vitro by the nanoformulations [46]. Nanosystems are internalized through endocytic processes that is time/energy dependent in contrast to the simple diffusion of the hydrophilic compound **1**. Furthermore, in case of SMA-**1** the internalized micelle needed to release its payload from the endocytic body (endosome, lysosome) to interact with the cytoplasmic HO-1 that is otherwise readily available to the free compound **1** [46,47]. The main advantage of nanoformulations is evident in in vivo systems, and it is due to the interaction between multiple organs and tissues, which results in an improved pharmacokinetics profile, such as prolonged T1/2, much slower elimination, and enhanced tumor accumulations [47]. Among all tested cell lines, B16 showed the highest response to HO-1 inhibition with compound **1** and good synergistic activity when administered in combination with doxorubicin 5 μM. This activity is consistent with previous reports demonstrating higher proliferation, stress resistance, higher antigenic activity, and poor survival span associated with HO-1 overexpression in this type of malignancies [44].

Overall, in vitro results showed that each tumor cell line responds differently to HO-1 inhibition, suggesting a differential expression and distinct roles in different cancers. The results suggest that HO-1 inhibition may be a convenient avenue in the management of some tumors, especially in patients with malignant melanoma. In addition the synergistic effect observed when 1 is administered in combination with doxorubicin suggests that HO-1 inhibition increase OS and consequently doxorubicin efficacy.

### 2.5. Docking Studies, ADME, and Toxicity Risk Assessment

**Figure 5:**
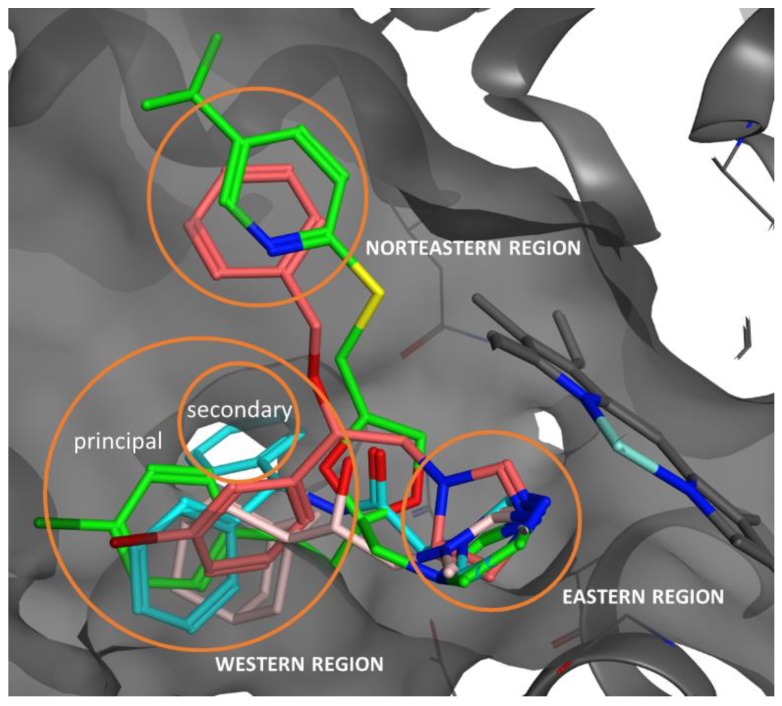
Docked poses of QC-80 (green), **2** (dark pink), **3** (light blue), and **4** (light pink).

In order to study the interaction of the new compounds **2**–**4** with HO-1, a molecular docking study was performed. The X-ray crystal structures of the co-crystal HO-1/QC-80 (PDB code 3HOK) was used as the protein structure. Docking was performed using AutoDock as described in the Materials and Methods [48]. To validate the docking model, we docked molecules QC-80 along with other classical inhibitors of HO-1 using the same validated docking procedure of our already published HO-1 inhibitors paper [21]. Once the model was validated, compounds **2**–**4** were studied in the docking experiment for HO-1, focusing the attention only on the (*S*)-enantiomers [21]. The docked poses and the 2D-interaction inside HO-1 are shown in Figure 5 and in Figures S3–S5 in the Supporting information. The results of the docking calculation are reported in Table 4. The calculated binding potencies are in good agreement with the experimental values in the HO-1 inhibition assay, as it is possible to see from a comparison between the calculated *K*_i_ and the experimental IC_50_. In the selected poses of the docked compounds, the iron(II) of the heme cofactor in HO-1 is correctly coordinated by the nitrogen atom of the imidazole ring of compounds **2**–**4** in the eastern pocket. By means of this coordination binding, iron(II) is protected from oxidation by disruption of an ordered solvent structure involving the critical Asp140 hydrogen-bond network (Tyr58, Tyr114, Arg136, and Asn210) and consequent displacement of water residues needed for catalysis. In the docked structures, the substituted phenylethanolic linker of our inhibitors is always located in the western region of the binding pocket, whereas the northeastern pocket is occupied by the benzyl substituent in the case of molecule **2**. Differently as expected by us, the benzylated analogue of compound **1**, compound **2**, does not allocate the benzyl group in the secondary binding pocket of the western region of HO-1; moreover, the benzyl group is located in the northeastern pocket (Asn210, Ala31, Ile211, Ala28 and Glu32, Figure S3) in a similar pose of the aromatic region (trifluoromethylpyridine) analogue in QC-80. Unfortunately, it was concluded that modification in this region would result in neither potency nor selectivity increases and may not be an efficient avenue in the development of highly selective HO-1 inhibitors [16]. This could be the reason for what compound **2** is around 200 fold less potent than the unsubstituted parent compound **1**. Differently, the bromophenyl moiety of the molecules is right allocated in the wester-principal region (Phe47, Val50, Phe167, Leu147, Leu54 and Arg136, Figure S3) and the bromine is pointing to the external portion inside the western region. With respect to compound **3**, it is well accommodated inside the binding pocket with a pose similar to that of the parent compound QC-308. Particularly, one of the two phenyl groups is located in the principal western pocket and the secondary western pocket is occupied by the second phenyl group (Ser53, Leu54, and Leu213, Figure S4), the nitrogen of the amide bond is involved in a hydrogen bonding interaction with Asp140. The aspartic acid in position 140 is also present in the isoform 2 of that protein (HO-2), and targeting this residue with a strong H-bond could be the reason for the lack in selectivity. In the case of compound **4**, the alcoholic function doesn’t get any stabilizing interaction, as previously described, and the adamantyl substituent is located deep inside the western-principal pocket (Phe166, Phe167, Leu54, Val50, Phe214 and Arg136, Figure S4).

To verify if the designed compounds showed a good pharmacokinetic profile and no adverse side effects (ADME-toxicity), we conducted an in silico study for compounds **2**–**4**. The results are reported in Table 5 and Table 6. The in silico ADME results (Table 5) clearly show that compounds **2**–**4** should exhibit a good oral availability (human intestinal absorption (HIA) > 95%) and a discrete Caco-2 cell permeability but with a strong plasma protein binding (PPB > 90%) in the case of compounds **2** and **3**, differently the calculated PPB for compound **4** is 67.75%. Interestingly, all of the compounds are supposed to discreetly permeate the blood-brain barrier (BBB) making them potential candidates for neuroblastoma therapy. Moreover, our new molecules resulted non-mutagen, non-tumorigenic, non-irritant and without negative effects on the reproductive system (Table 6). Finally, compounds **2**–**4** have a positive value of drug-likeness, establishing that the molecule predominantly contains common fragments that are present in commercial drugs, and a drug-score, that encompass the contributions of partition coefficient, solubility, molecular weight, drug-likeness, and the four toxicity risks, higher than that of the compounds so far reported [21].

## 3. Materials and Methods

### 3.1. Chemistry

Melting points were determined in an IA9200 Electrothermal apparatus equipped with a digital thermometer in glass capillary tubes and are uncorrected. Infrared spectra were recorded on a Perkin Elmer 281 FTIR spectrometer in KBr disks or NaCl crystal windows. Elemental analyses for C, H, N, and S were within ±0.4% of theoretical values and were performed on a Carlo Erba Elemental Analyzer Mod. 1108 apparatus. ^1^H NMR spectra were recorded on a Varian Unity Inova 500 MHz spectrometer in DMSO-*d*_6_ solution. Chemical shifts are given in *δ* values (ppm), using tetramethylsilane (TMS) as the internal standard; coupling constants (*J*) are given in Hz. Signal multiplicities are characterized as s (singlet), d (doublet), t (triplet), q (quartet), m (multiplet), br (broad). All the synthesized compounds were tested for purity on TLC (aluminum sheet coated with silica gel 60 F254, Merck, Kenilworth, NJ, USA) and visualized by UV (λ = 254 and 366 nm). Purification of synthesized compounds by column chromatography was performed using silica gel 60 (Merck, Kenilworth, NJ, USA). All chemicals and solvents were reagent grade and were purchased from commercial vendors.

#### 3.1.1. 1-[2-(3-Bromophenyl)-2-(phenylmethoxy)ethyl]-1*H*-imidazole (**2**)

A suspension of compound **1** (133.6 mg, 0.5 mmol) and NaH 80% dispersion in mineral oil (0.65 mmol, 19.5 mg) dissolved in 2 mL of dry DMF was added dropwise with a solution of benzyl bromide in 1 mL of dry DMF. The mixture was left stirring for 2 h, added with 5 mL of methanol and concentrated to reduced volume under vacuum. The residue was diluted with water, washed with EtOAc (3 × 50 mL). The combined organic layers were washed with brine (1 × 100 mL), dried over anhydrous Na_2_SO_4_, filtered, and evaporated. The obtained crude material was purified by column chromatography using EtOAc (100%) as eluent, to afford the title compound as pure yellowish oil (yield 82%); IR (NaCl, selected lines) cm^−1^ 3387, 3110, 2932, 1570, 1507, 1474, 1285, 1231, 1106, 1074, 1028, 737, 697; ^1^H NMR (500 MHz, DMSO-*d*_6_): 7.59–7.51 (m, 2H + 1H, aromatic + imidazole), 7.39–7.25 (m, 5H, aromatic), 7.18–7.08 (m, 2H + 1H, aromatic + imidazole), 6.87 (s, 1H, imidazole), 4.79–4.72 (m, 1H, CH), 4.41–4.20 (m, 2H + 2H, CH_2_ + CH_2_); ^13^C NMR (125 MHz, DMSO-*d*_6_) 142.14, 138.31, 131.46, 131.19, 130.05, 128.70, 128.41, 127.91, 127.57, 126.18, 122.32, 120.50, 79.90, 70.71, 52.04. Anal. calcd. for C_18_H_17_BrN_2_O (%) C, 49.46; H, 4.15; N, 10.49. Found: C49.78; H, 4.02; N, 10.81.

#### 3.1.2. *N*-Benzhydryl-2-(1*H*-imidazol-1-yl)acetamide (**3**)

*N*-Benzhydryl-2-bromoacetamide (500 mg, 1.64 mmol) was dissolved in anhydrous DMF (5 mL) and added dropwise to a previously prepared suspension of imidazole (4.93 mmol) and K_2_CO_3_ (4.93 mmol) in anhydrous DMF (5 mL). The obtained reaction mixture was left stirring for 2 h, then, water was added and the resulting suspension was filtered. The crude filtrate was purified by crystallization using a mixture of EtOH/water (1:1) to give 128 mg of the title compound as a whitish solid (yield 27%): m.p. 196–197 °C; IR (KBr, selected lines) cm^−1^ 3252, 3263, 1657, 1554, 1508, 1493, 1292, 1267, 1230, 1078, 760, 745; ^1^H NMR (500 MHz, DMSO-*d*_6_): 9.13 (d, *J* = 8.5 Hz, 1H, NH), 7.58 (s, 1H, imidazole), 7.38–7.24 (m, 10H, aromatic), 7.10 (s, 1H, imidazole), 6.86 (s, 1H, imidazole), 6.11 (d, *J* = 8.5 Hz, 1H, CH), 4.80 (s, 2H, CH_2_); ^13^C NMR (125 MHz, DMSO-*d*_6_) 166.13, 142.07, 128.41, 127.91, 127.20, 127.06, 120.43, 56.22, 48.58; Anal. calcd. for C_18_H_17_N_3_O (%) C, 74.20; H, 5.88; N, 14.42. Found: C, 74.38; H, 5.82; N, 14.71.

#### 3.1.3. 2-(1*H*-Imidazol-1-yl)-1-tricyclo[3.3.1.13,7]dec-1-yl-ethanol (**4**)

A mixture of QC-82 (2.0 mmol) and NaBH_4_ (2.2 mmol) in anhydrous methanol (10 mL) was refluxed for 2 h, then it was evaporated to dryness, added with 40 mL of deionized water, acidified with HCl 2N, and heated to 110 °C for 0.5 h. After cooling to room temperature, the reaction mixture was treated with NaOH 0.5 N up to a pH = 8–9 and the obtained suspension was filtered, washed repeatedly with water to neutrality, and dried. Recrystallization from EtOH gave the title compound as a pure white solid (yield 89%): m.p. 182–183 °C; IR (KBr, selected lines) cm^−1^ 3184, 3112, 2904, 1516, 1450, 1343, 1234, 1106, 1078, 921, 747; ^1^H NMR (500 MHz, DMSO-*d*_6_): 7.57 (s, 1H, imidazole), 7.13 (s, 1H, imidazole), 6.84 (s, 1H, imidazole), 4.79 (d, *J* = 6.5 Hz, 1H, OH), 4.15–4.06 (m, 1H, C*H*_A_H_B_N), 3.70 (dd, *J* = 10 Hz, *J* = 14 Hz, 1H, CH_A_*H*_B_N), 3.09–3.01 (m, 1H, CHOH), 1.99–1.89 (m, 3H, adamantane), 1.70–1.49 (m, 12H, adamantane); ^13^C NMR (125 MHz, DMSO-*d*_6_) 138.22, 128.24, 120.39, 78.39, 48.25, 38.05, 37.23, 36.53, 28.20. Anal. calcd. for C_15_H_22_N_2_O (%) C, 73.13; H, 9.00; N, 11.37. Found: C, 73.25; H, 8.71; N, 11.09.

### 3.2. Synthesis of SMA-***1*** Micelles

SMA micelles were prepared as described previously [49]. SMA was hydrolyzed by dissolving the powder in 1.0 M NaOH at 70 °C to achieve a concentration of 10 mg/mL. Hydrolyzed SMA was then adjusted to pH 5.0 and *N*-ethyl-*N*-(3-dimethylaminopropyl) carbodiimide hydrochloride (EDAC) added in a 1:1 ratio by weight to SMA. SMA (80 mg) was dissolved in 50 mL of deionized water (DW), EDAC 80 mg dissolved in 20 mL of DW, Compound **1** (20 mg) dissolved in 5 mL of DMSO. A solution of 1 was added to the SMA solution simultaneously and the pH was maintained at 5.0 until stabilized. Once the pH stabilized at 5.0, the pH was raised to 11.0 and maintained stable, then the pH was lowered to 7.4 and the solution was filtered four times using a Millipore Lab scale TFF system (Merck Millipore, Burlington, MA, USA) with a Pellicon XL (EMD Millipore, Burlington, MA, USA) 10 kDa cut-off membrane. The solution was frozen at −80 °C overnight before being lyophilized to obtain the SMA-**1** micelles powder.

#### 3.2.1. Loading of the SMA-**1** Micelles

Compound **1** loading into the SMA micelles was determined by arranging triplicate samples of SMA-**1** micelles in DMSO at a concentration of 1.0 mg/mL and then reading and comparing the absorbance of the drug to a pre-prepared standard curve (Figure S1, supporting information) of the free drug at 272 nm in order to determine the weight ratio of the loaded drug.

#### 3.2.2. Size, PDI, and Zeta Potential Determination of SMA Micelles

Lyophilized SMA-**1** micelles were solubilized in either NaHCO_3_ (0.1 M, pH 7.8) to determine the size and PDI or in distilled water to estimate the charge. All measurements were done using the Malvern ZEN3600 Zetasizer Nano series (Malvern Instruments, Malvern, UK) (Figure S2, supporting information). The results were obtained from three independent experiments.

### 3.3. Biology

#### 3.3.1. Preparation of Spleen and Brain Microsomal Fractions

HO-1 and HO-2 were obtained from rat spleen and brain, respectively, as the microsomal fraction prepared by differential centrifugation; the dominance of HO-1 protein in the rat spleen and of HO-2 in the rat brain has been well documented [50,51,52,53]. These particular microsomal preparations were selected in order to use the most native (i.e., closest to in vivo) forms of HO-1 and HO-2. Spleen and brain (Sprague–Dawley rats) microsomal fractions were prepared according to the procedure outlined by Ryter et al. [54]. The experiments reported in the present paper complied with current Italian law and met the guidelines of the Institutional Animal Care and Use Committee of University of Catania (Italy). The experiments were performed in male Sprague–Dawley albino rats (150 g body weight and age 45 d). They had free access to water and were kept at room temperature with a natural photo-period (12 h light-12 h dark cycle). For measuring HO-1 and HO-2 activities, each rat was sacrificed and their spleen and brain were excised and weighed. A homogenate (15%, *w*/*v*) of spleens and brains pooled from four rats was prepared in ice-cold HO-homogenizing buffer (50 mM Tris buffer, pH 7.4, containing 0.25 M sucrose) using a Potter–Elvehjem homogenizing system with a Teflon pestle. The microsomal fraction of rat spleen and brain homogenate was obtained by centrifugation at 10,000 rpm for 20 min at 4 °C, followed by centrifugation of the supernatant at 100,000 rpm for 60 min at 4 °C. The 100,000 rpm pellet (microsomes) was resuspended in 100 mM potassium phosphate buffer, pH 7.8, containing 2 mM MgCl_2_ with a Potter–Elvehjem homogenizing system. The rat spleen and brain microsomal fractions were divided into equal aliquots, placed into microcentrifuge tubes, and stored at −80 °C for up to 2 months. Protein concentration of the microsomal fraction was determined by Lowry method [55].

#### 3.3.2. Preparation of Biliverdin Reductase

Liver cytosol has been used as a source of biliverdin reductase (BVR). Rat liver was perfused through the hepatic portal vein with cold 0.9% NaCl, and then it was cut and flushed with 2 × 20 mL of ice-cold PBS to remove all of the blood. Liver tissue was homogenized in three volumes of a solution containing 1.15% KCl *w*/*v* and Tris buffer 20 mM, pH 7.8 on ice. Homogenates were centrifuged at 10,000 rpm, for 20 min at 4 °C. The supernatant was decanted and centrifuged at 100,000 rpm for 1 h at 4 °C to sediment the microsomes. The 100,000 rpm supernatant was saved and then stored in small amounts at −80 °C after its protein concentration was measured.

#### 3.3.3. Measurement of HO-1 and HO-2 Enzymatic Activities in Microsomal Fraction of Rat Spleen and Brain

The HO-1 and HO-2 activities were determined by measuring the BR formation using the difference in absorbance at 464 to 530 nm as described by Ryter et al. [54]. Reaction mixtures (500 μL) consisted of 20 mM Tris-HCl, pH 7.4, (1 mg/mL) microsomal extract, 0.5–2.0 mg/mL BVR, 1 mM NADPH, 2 mM glucose 6-phosphate (G6P), 1 U G6P dehydrogenase, 25 μM hemin, and 10 μL of DMSO (or the same volume of DMSO solution of test compounds to a final concentration of 100, 10, and 1 μM). Incubations were carried out for 60 min at 37 °C in a circulating water bath in the dark. Reactions were stopped by adding 1 volume of chloroform. After recovering the chloroform phase, the amount of BR formed was measured with a double-beam spectrophotometer as OD_464-530_ nm (extinction coefficient, 40 mM/cm^−1^ for BR). One unit of the enzyme was defined as the amount of enzyme catalyzing the formation of 1 nmol of BR/mg protein/h.

#### 3.3.4. Cell Cultures

MDA-MB-231 cells (hormone resistant), MCF-7 (hormone sensitive), PC3 cells (hormone resistant), DU145 cells (hormone resistant), LnCap cells (hormone sensitive), B16, and HEK cells were maintained in complete growth media (DMEM/Ham’s F12 supplemented with 10% fetal bovine serum, 2 mL-glutamine, 100 units/mL penicillin, 100 units/mL of streptomycin, and 2.2 g/L of NaHCO_3_) at 37 °C in a humidified atmosphere of 5% CO. For all procedures, cells were harvested using TrypLE Express (Life Technologies, Auckland, New Zealand).

#### 3.3.5. In Vitro Cytotoxicity of HO Inhibitors against Breast and Prostate Cancer Cell Lines, Murine Melanoma, and HEK Cells

To test the cytotoxic effect of HO inhibitors, cells (8 × 103 cells/well) were seeded into 96-well plates and incubated for 24 h. This was followed by treatment with HO inhibitors at the concentrations 1.1–200 μM (1.1–1000 μM for HEK cells) of compound **1**. In addition, the cytotoxicity of doxorubicin (5 μM, 10 μM, and 30 μM) and a combination of doxorubicin 5 μM and 37 μM **1** was assessed using B16 cells. In all experiments, the cells were incubated for 72 h. Following the incubation, cells were fixed using 10% trichloroacetic acid (TCA). Cytotoxicity was determined using the sulforhodamine B assay as previously described [56]. The concentration required to decrease the cell number by 50% (IC_50_) was determined by non-linear regression using GraphPad Prism 6 software (version 7.0, GraphPad Software Inc., San Diego, CA, USA). Treatments were performed in triplicate and data represents mean of three independent experiments.

### 3.4. Docking

#### 3.4.1. Preparation of Ligands

The 3D structures of ligands were built using Gabedit (2.4.8) software (Gabedit software 2.4.8, Abdul-Rahman Allouche, Lyon, France) [57] and all geometries were fully optimized, in the same software, with the semi-empirical PM6 Hamiltonian [58] implemented in MOPAC2016 (17.130 W) [59].

#### 3.4.2. Docking Protocol

Macromolecules and ligands were prepared within YASARA; the point charges were initially assigned according to the AMBER14 force field [60], and then damped to mimic the less polar Gasteiger charges used to optimize the AutoDock scoring function. Fine docking was performed by applying the Lamarckian genetic algorithm (LGA) implemented in AutoDock. The ligand-centered maps were generated by the program AutoGrid with a spacing of 0.375 Å and dimensions that encompass all atoms extending 5 Å from the surface of the ligand. All of the parameters were inserted at their default settings. In the docking tab, the macromolecule and ligand are selected, and GA parameters are set as ga_runs = 100, ga_pop_size = 150, ga_num_evals = 20,000,000, ga_num_generations = 27,000, ga_elitism = 1, ga_mutation_rate = 0.02, ga_crossover_rate = 0.8, ga_crossover_mode = two points, ga_cauchy_alpha = 0.0, ga_cauchy_beta = 1.0, number of generations for picking worst individual = 10.

From the crystal structures of the HO-1/QC-80 (PDB code 3HOK) complex, we retained only the chain B and the prosthetic-heme group. Because no water molecules are directly involved in complex stabilization they were not considered in the docking process. All protein amino acidic residues were kept rigid whereas all single bonds of ligands were treated as full flexible.

## 4. Conclusions

In this study, we report the design, synthesis, and biological properties of three new imidazole-based HO-1 inhibitors, compounds **2**–**4**, and the in vitro anticancer activity profile of a previous compound synthesized by us, compound **1**. Differently to molecule **1**, which is one of the most potent/selective HO-1 inhibitors known to date with sub-micromolar inhibitory activity against HO-1 and moderate activity against HO-2 (HO-1 IC_50_ = 0.4 µM, HO-2 IC_50_ = 32.0 µM), the new compounds **2**–**4** were still active but possess IC_50_ values in the micromolar range, IC_50_ = 80, 28.8, and 67.6 µM, respectively. Modeling studies of these three new imidazole-based inhibitors proved that in the molecules **2**–**4**, the imidazole interacted with the iron of the heme group. All hydrophobic groups are allocated in the principal western pocket. The northeastern pocket was occupied by the benzyl substituent in the case of molecule **2** and the secondary western pocket is occupied by one phenyl of molecule **3**. Moreover, in silico prediction of the ADME-Tox profiles for the three compounds highlighted that they exhibited a good oral availability, a strong plasma protein binding, and a discrete capacity to permeate the BBB. Furthermore, molecules **2**–**4** resulted non-mutagen, non-tumorigenic, non-irritant, and without negative effects on the reproductive system, possessing an elevated value of drug-score, suggesting them as the new lead candidates for further studies. The in vitro anticancer activity profile of compound **1** was investigated in different cancer cell lines and in HEK cells. Compound **1** showed the most interesting results for melanoma (B16 IC_50_ = 42 μM) and hormone-sensitive cell lines (MCF-7 IC_50_ = 52.55 μM) when compared to hormone-resistant (MDA-MB-231 IC_50_ = 82.60 μM) and showed certain selectivity towards normal cells. Moreover, combination of compound **1** and doxorubicin give synergistic activity in melanoma cell lines. Finally, considering the micelles formulation as an efficient way to solubilize/carry hydrophobic drugs and an elegant example of supramolecular structures for its use in drug delivery and as smart systems for efficient targeting, a nanomicellar formulation was synthesized for compound **1**. The formulation was also tested for its cytotoxicity with reduced cytotoxic activity.

## Figures and Tables

**Table 1:** Characterization of SMA-**1** ^a^.

Micelle	Recovery	Loading (*wt*/*wt*)	Size (nm)	PDI	Zeta Potential (mV)
SMA-**1**	80%	18%	180.6 ± 12.3	0.211	−0.11

^a^ Data are shown as mean values ± standard deviation (SD). Values are the mean of triplicate experiments. PDI = polydispersity index.

**Table 2:** Inhibitory potency of compounds **1**–**4** towards HO-1 and HO-2.

Compound	IC_50_ (μM) ^a^	
	HO-1	HO-2
(*R*/*S*)-**1**	0.40 ± 0.01 ^b^	32.0 ± 2.2 ^b^
(*R*/*S*)-**2**	80.0 ± 3.3	ND ^c^
**3**	28.8 ± 1.4	14.4 ± 0.9
(*R*/*S*)-**4**	67.6 ± 2.1	ND ^c^
Azalanstat ^b^	5.3 ± 0.4	24.4 ± 0.8

^a^ Data are shown as IC_50_ values in μM ± standard deviation (SD). Values are the mean of triplicate experiments; ^b^ Reported in Reference [21]; ^c^ ND = not determined.

**Table 3:** IC_50_ values for HO-1 inhibitor compounds in hormone resistant and hormone-responsive breast and prostate cancer cells, murine melanoma, and in human embryonic kidney (HEK) cells.

Compound	IC_50_ (μM) ^a^						
	MDA-MB-231	MCF-7	DU145	PC3	LnCap	B16	HEK
**1**	82.60 ± 0.57	52.55 ± 3.76	137.60 ± 2.7	164.73 ± 2.8	158.03 ± 2.3	37.00 ± 7.25	363.5 ± 21.3
SMA-**1**	>100	>100	>200	>200	>200	>100	>300

^a^ Data are shown as IC_50_ values in μM ± standard deviation (SD). Values are the mean of triplicate experiments.

**Table 4:** Docking results for molecules (*S*)-2, 3, (*S*)-4.

Compound	*ΔG_B_* Calcd. (kcal/mol)	*K*_i_ Calcd. (μM)	Exp. IC_50_ (μM) HO-1
**2**	−6.24	26.5	80.0
**3**	−7.04	6.87	28.8
**4**	−6.75	11.2	67.6

**Table 5:** Selected in silico ADME profiling for molecules **2** and **3** ^a^.

Compound	Absorption		Distribution	
	HIA (%)	In Vitro Caco-2 Cell Permeability (nm s^−1^)	In Vitro PPB (%)	In Vivo BBB Penetration (C_brain_/C_blood_)
**2**	100.0	57.31	94.39	2.01
**3**	95.86	36.43	100	0.57
**4**	95.30	26.33	67.75	0.77

^a^ The properties related to ADME were predicted using PreADMET web-based application (http://preadmet.bmdrc.kr).

**Table 6:** In silico toxicity parameters for molecules **2** and **3** ^a^.

Compound	Mutagenic	Tumorigenic	Reproductive Effects	Irritant	Drug-Likeness	Drug-Score
**2**	none	none	None	none	2.90	0.78
**3**	none	none	None	none	5.03	0.91
**4**	none	none	None	none	5.10	0.93

^a^ The properties related to toxicity were predicted using DataWarrior software.

## Supplementary Materials

The following materials are available online, Figure S1: Pre-prepared calibration curve of the free compound **1** at 272 nm; Figure S2: Size distribution of SMA-**1**; Figure S3: 2D interaction and docked pose of compound **2**; Figure S4: 2D interaction and docked pose of compound **3**; Figure S5: 2D interaction and docked pose of compound **4**. NMR spectra of compounds **2**–**4**.
