# Supplementary material for: Novel Structural Insight into Inhibitors of Heme Oxygenase-1 (HO-1) by New Imidazole-Based Compounds: Biochemical and In Vitro Anticancer Activity Evaluation
Source: Molecules. 2018 May 18;23(5):1209. doi: 10.3390/molecules23051209 (PMC6099553; doi:10.3390/molecules23051209)
Supplement: Supplementary file 1 [file molecules-23-01209-s001.pdf]

molecules

Supplementary Informations

Novel structural insight into inhibitors of Heme Oxygenase-1 (HO-1) by new imidazole-based compounds: biochemical and in vitro anticancer activity evaluation

Khaled F. Greish [1], Loredana Salerno [2,*], Reem Al Zahrani [1], Emanuele Amata [2], Maria N. Modica [2], Giuseppe Romeo [2], Agostino Marrazzo [2], Orazio Prezzavento [2], Valeria Sorrenti [2], Antonio Rescifina [2], Giuseppe Floresta [2,3,*], Sebastiano Intagliata [4], Valeria Pittalà [2]

[1] Department of Molecular Medicine, College of Medicine and Medical Sciences, Princess Al-Jawhara Centre for Molecular Medicine, Arabian Gulf University, Manama, Kingdom of Bahrain
[2] Department of Drug Sciences, University of Catania, V.le A. Doria 6, 95125 Catania, Italy
[3] Department of Chemical Sciences, University of Catania, V.le A. Doria, 95125 Catania, Italy
[4] Department of Medicinal Chemistry, College of Pharmacy, University of Florida, Gainesville, FL, USA
* Correspondence: giuseppe.floresta@unict.it (G.F.) and l.salerno@unict.it (L.S.); Tel.: +39-095-738-4266

Received: date; Accepted: date; Published: date

Table of contents

Calibration curve of the free compound **1** at 272 nm (Figure S1) S2
Size distribution of SMA-**1** (Figure S2) S2
2D interaction and docked pose of compound **2** (Figure S3) S3
2D interaction and docked pose of compound **3** (Figure S4) S3
2D interaction and docked pose of compound **4** (Figure S5) S4
NMR spectra of compounds **2–4** **S**5

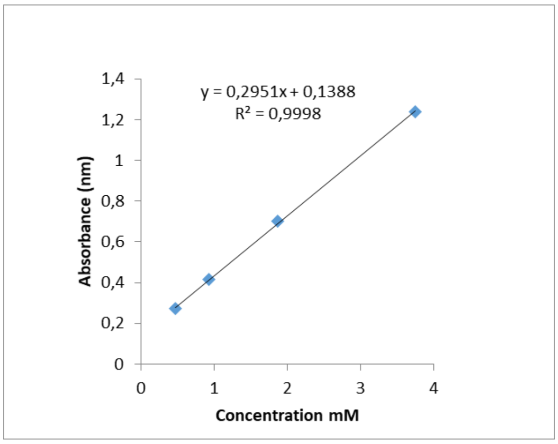

Figure S1. Calibration curve of the free compound 1 at 272 nm.

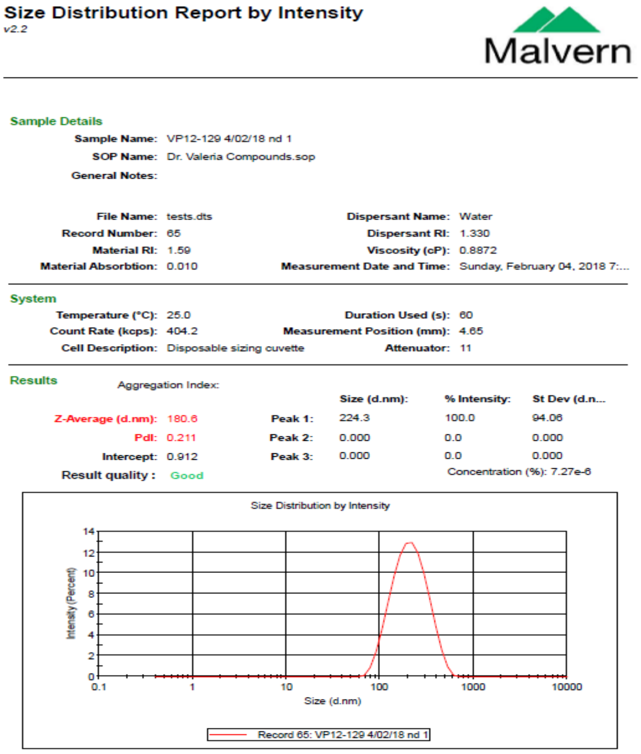

Figure S2. Size distribution of SMA-**1** (the micelles are identified in the picture as VP12-129).

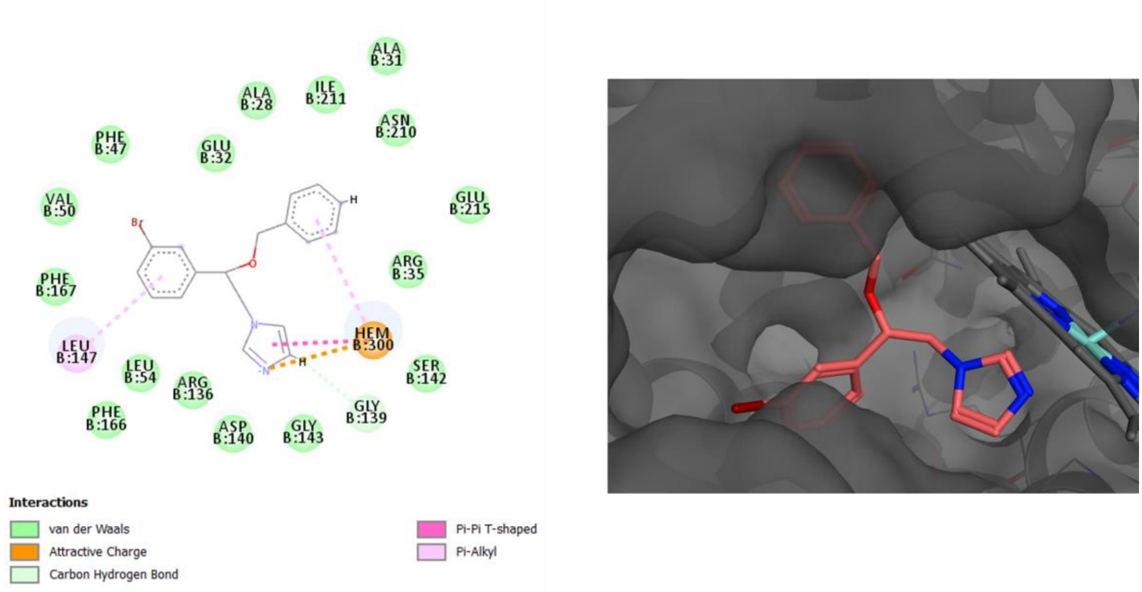

Figure S3. 2D interaction and docked pose of compound **2**.

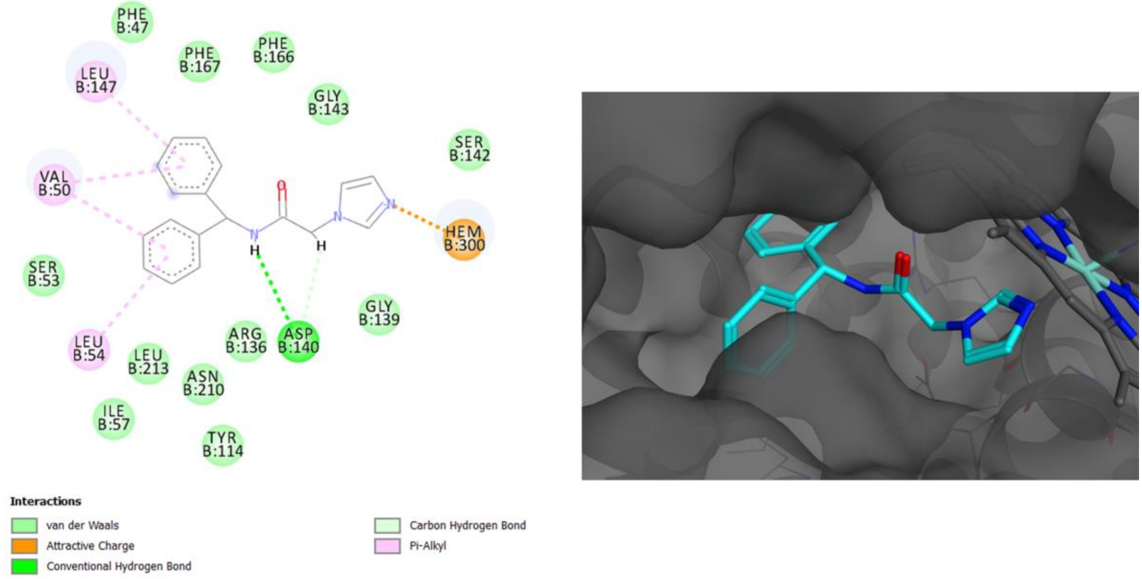

Figure S4. 2D interaction and docked pose of compound **3**.

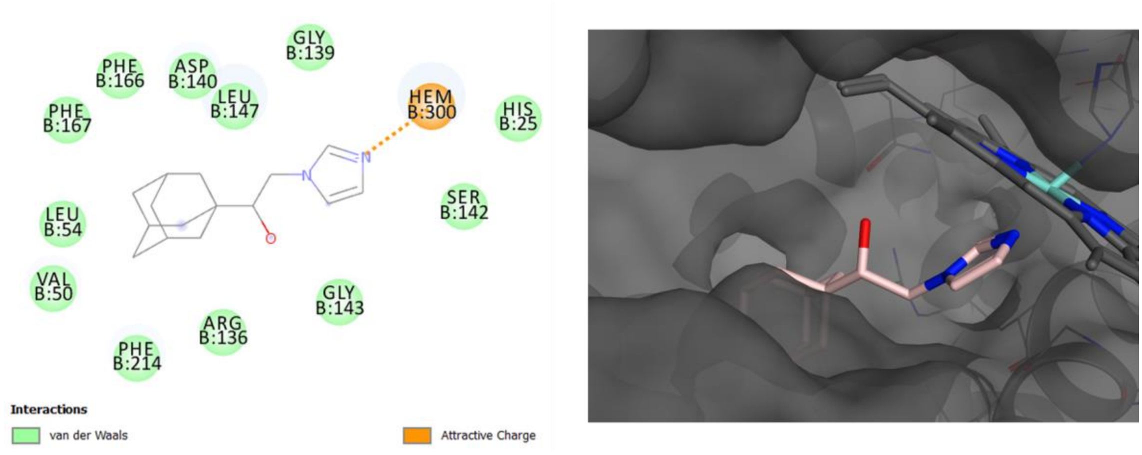

Figure S5. 2D interaction and docked pose of compound **4**.

^{1}H NMR of compound 2

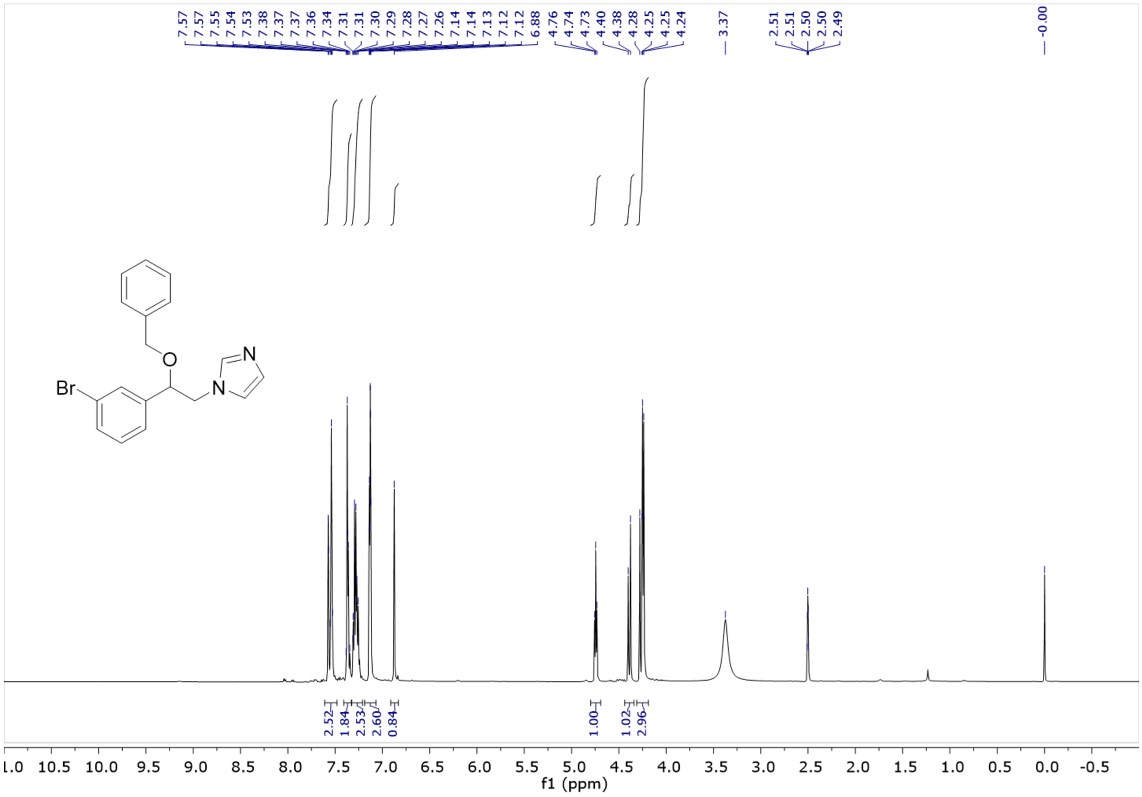

^{13}C NMR of compound 2

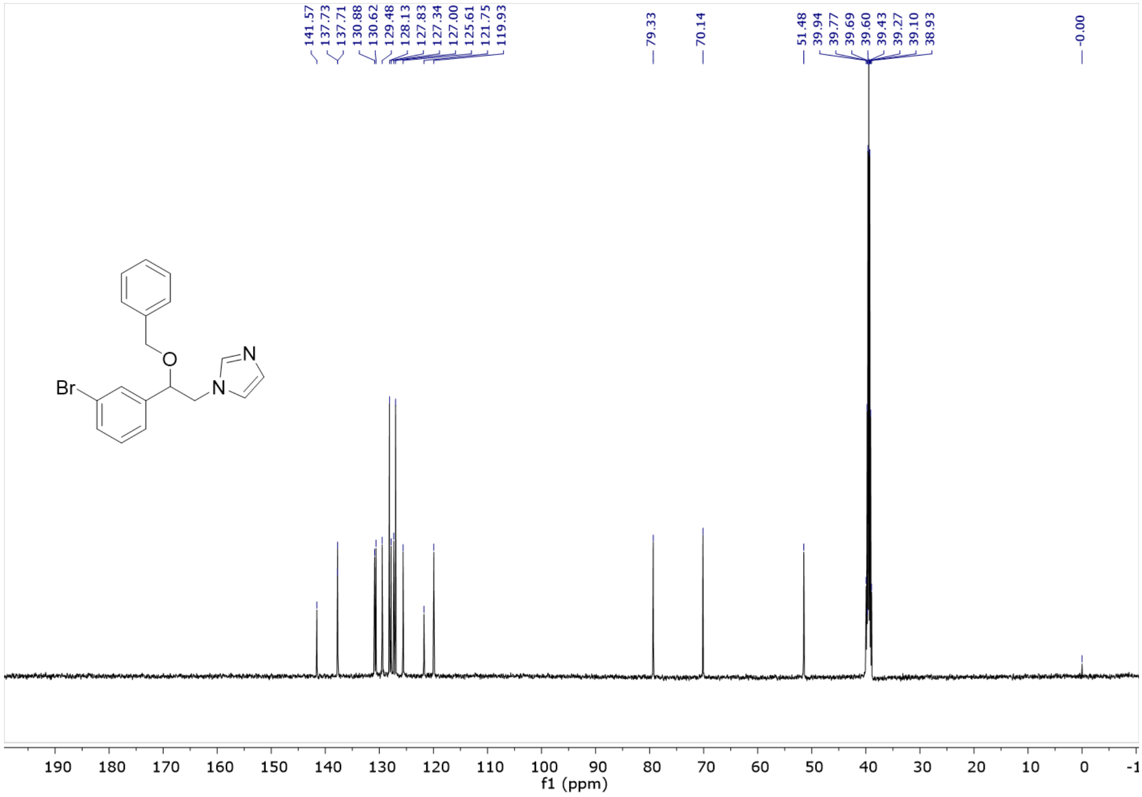

^{1}H NMR of compound 3

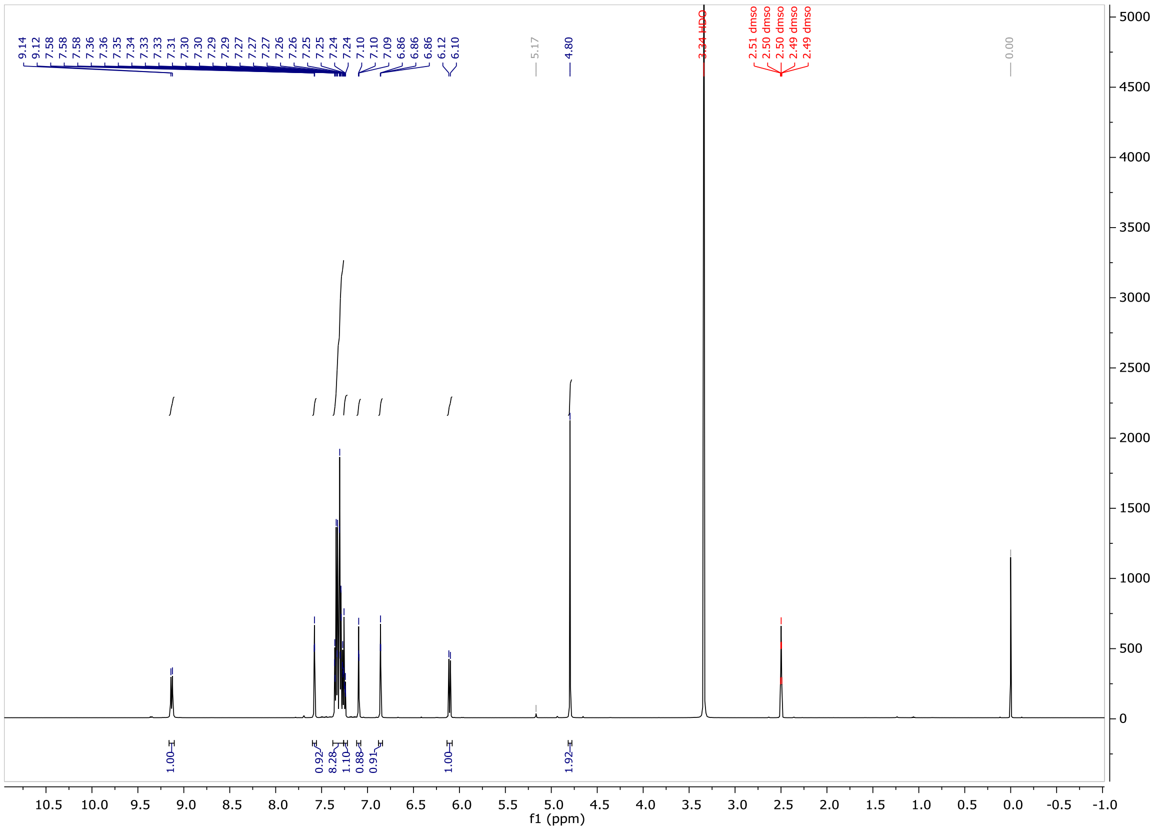

^{13}C NMR of compound 3

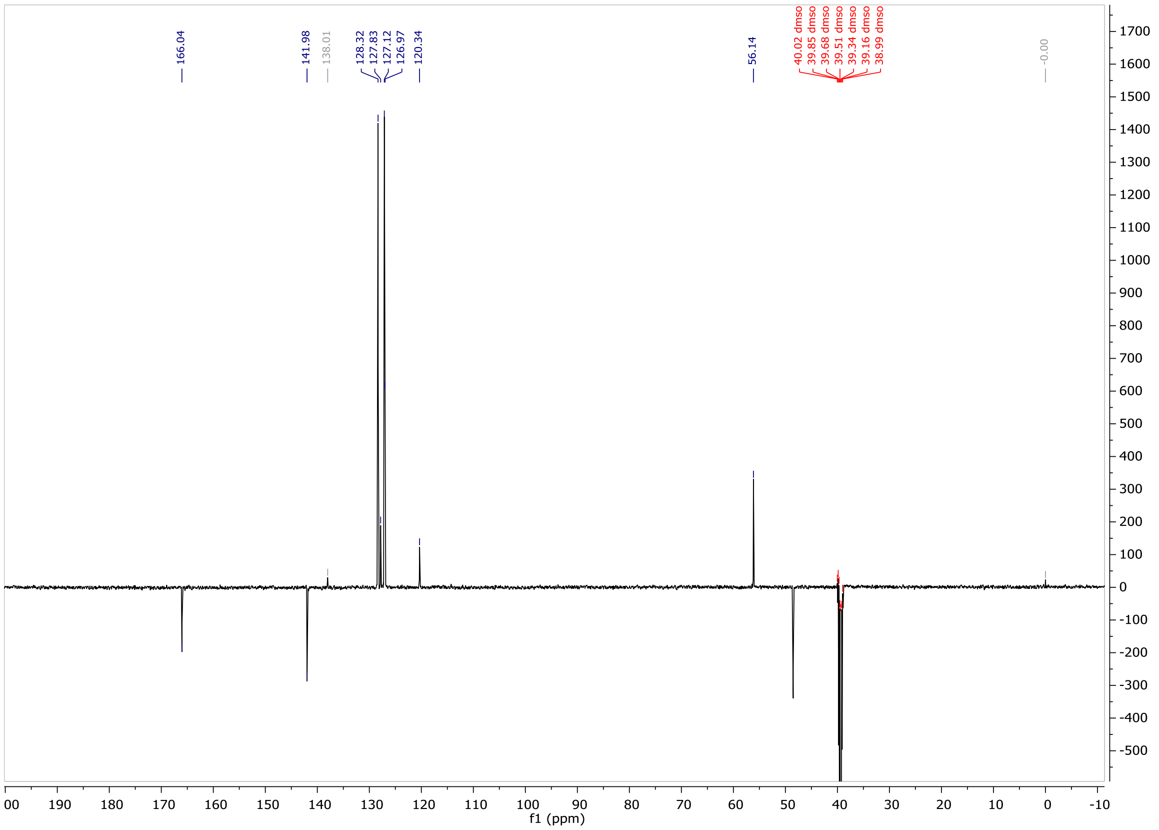

^{1}H NMR of compound 4

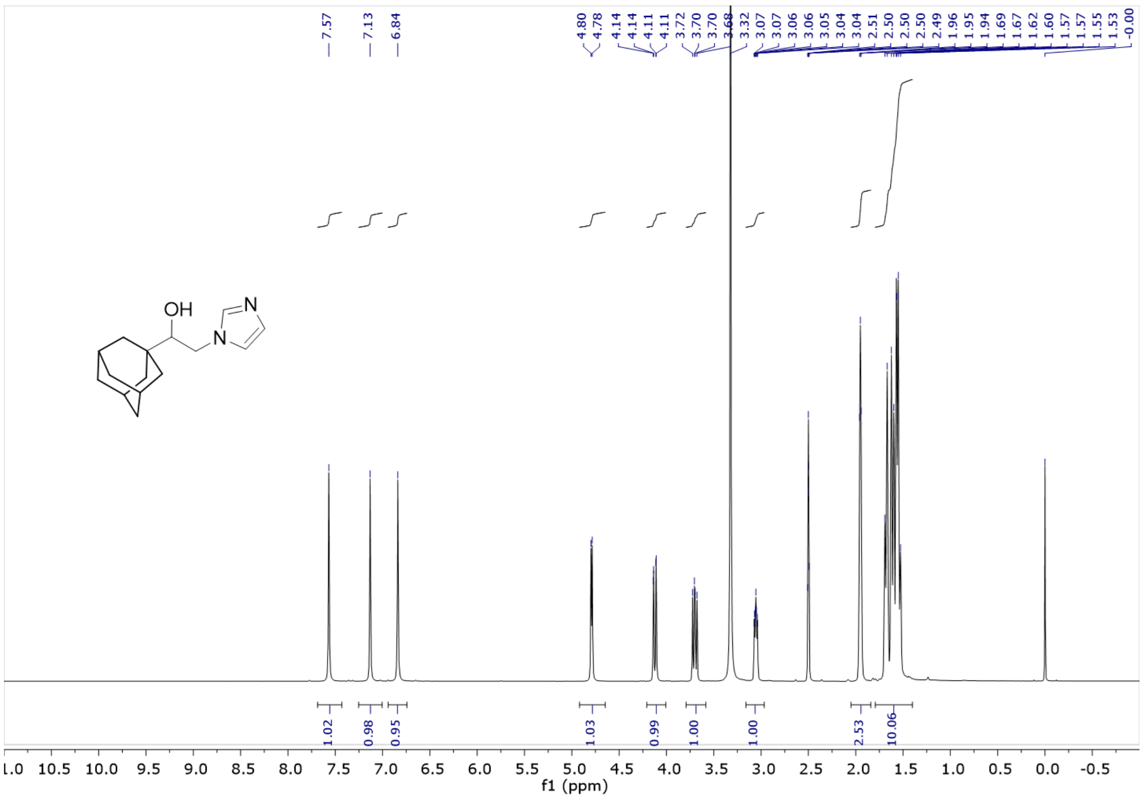

^{13}C NMR of compound 4

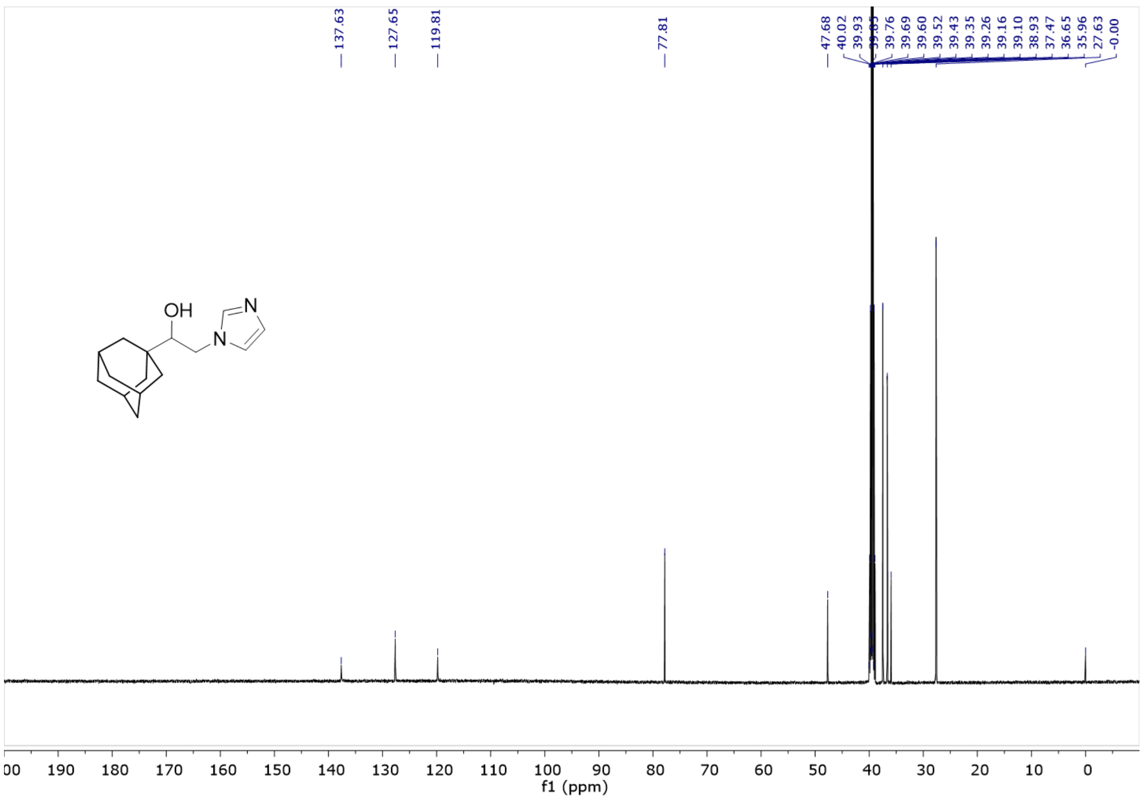

© 2018 by the authors. Submitted for possible open access publication under the terms and conditions of the Creative Commons Attribution (CC BY) license (http://creativecommons.org/licenses/by/4.0/).
